# Herbal Medicines Targeting the Improved *β*-Cell Functions and *β*-Cell Regeneration for the Management of Diabetes Mellitus

**DOI:** 10.1155/2021/2920530

**Published:** 2021-07-14

**Authors:** Akurange Sujeevi Dammadinna Wickramasinghe, Pabasara Kalansuriya, Anoja Priyadarshani Attanayake

**Affiliations:** ^1^Department of Pharmacy, Faculty of Health Sciences, The Open University of Sri Lanka, Nawala, Sri Lanka; ^2^Department of Biochemistry, Faculty of Medicine, University of Ruhuna, Galle, Sri Lanka

## Abstract

There is an increasing trend of investigating natural bioactive compounds targeting pancreatic *β*-cells for the prevention/treatment of diabetes mellitus (DM). With the exploration of multiple mechanisms by which *β*-cells involve in the pathogenesis of DM, herbal medicines are gaining attention due to their multitasking ability as evidenced by traditional medicine practices. This review attempts to summarize herbal medicines with the potential for improvement of *β*-cell functions and regeneration as scientifically proven by *in vivo/in vitro* investigations. Furthermore, attempts have been made to identify the mechanisms of improving the function and regeneration of *β*-cells by herbal medicines. Relevant data published from January 2009 to March 2020 were collected by searching electronic databases “PubMed,” “ScienceDirect,” and “Google Scholar” and studied for this review. Single herbal extracts, polyherbal mixtures, and isolated compounds derived from approximately 110 medicinal plants belonging to 51 different plant families had been investigated in recent years and found to be targeting *β*-cells. Many herbal medicines showed improvement of *β*-cell function as observed through homeostatic model assessment-*β*-cell function (HOMA-*β*). Pancreatic *β*-cell regeneration as observed in histopathological and immunohistochemical studies in terms of increase of size and number of functional *β-*cells was also prominent. Increasing *β-*cell mass via expression of genes/proteins related to antiapoptotic actions and *β-*cell neogenesis/proliferation, increasing glucose-stimulated insulin secretion via activating glucose transporter-2 (GLUT-2) receptors, and/or increasing intracellular Ca^2+^ levels were observed upon treatment of some herbal medicines. Some herbal medicines acted on various insulin signaling pathways. Furthermore, many herbal medicines showed protective effects on *β-*cells via reduction of oxidative stress and inflammation. However, there are many unexplored avenues. Thus, further investigations are warranted in elucidating mechanisms of improving *β-*cell function and mass by herbal medicines, their structure-activity relationship (SAR), and toxicities of these herbal medicines.

## 1. Introduction

DM is a chronic metabolic disorder characterized by hyperglycemia which is caused by deficiency of insulin secretion, insulin action, or both [1]. DM has become a major global health issue since it causes premature mortality and lower quality of life due to diabetes-related complications. According to the latest data provided by the International Diabetes Federation, the global adult population (20–79 years) with DM was approximately 463 million (9.3% of the global adult population) in the year 2019 and is estimated to rise up to 700 million by the year 2045. DM and its complications caused 4.2 million deaths worldwide in the year 2019. The prevalence of DM among the Sri Lankan adult population is reported as 8.7% [2]. DM accounts for 9% mortality of all deaths in Sri Lanka [3]. The majority of DM cases can be categorized into two broad categories, i.e., type 1 diabetes mellitus (T1DM) and type 2 diabetes mellitus (T2DM). T1DM is caused due to autoimmune *β-*cell destruction which leads to an absolute deficiency of insulin secretion whereas T2DM resulted from a combination of resistance to insulin and inadequate compensatory insulin secretion [4]. At present, around 90% of diabetic cases are of T2DM with the highest proportion in low and middle-income countries [2].

Pancreatic *β-*cells which secrete the anabolic hormone insulin have an important role in glucose homeostasis hence in the pathogenesis of DM [5]. Insulin is a polypeptide consisting of two chains of amino acids known as A and B chains, linked by three disulfide bridges. Initially, a peptide chain known as preproinsulin is synthesized by mRNA coded for insulin which gets converted to proinsulin in the endoplasmic reticulum. Proinsulin is then shifted to the Golgi apparatus and assembled into granules. The peptide segment connecting the A and B chains, the connecting peptide (C-peptide), is detached in the granules before insulin secretion making equimolar amounts of insulin and C-peptide in the granules. C-peptide levels can be measured, and its level in the blood provides an index of *β-*cell function in patients receiving exogenous insulin [6].

**Figure 1:**
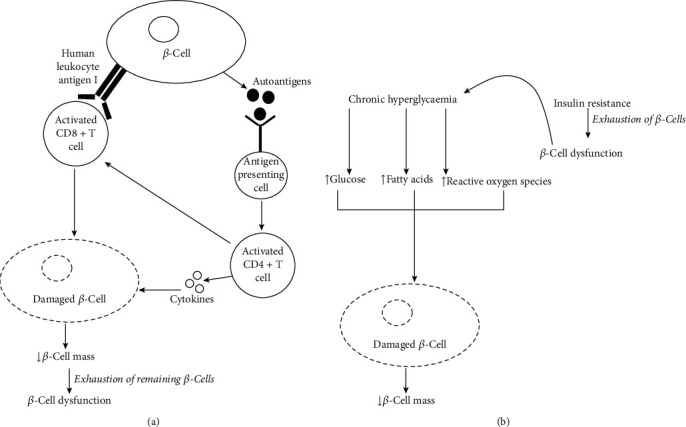
An overview of mechanisms involved in *β-*cell damage and dysfunction in (a) type 1 diabetes mellitus and (b) type 2 diabetes mellitus.

An overview of mechanisms involved in *β-*cell damage and dysfunction in the pathogenesis of T1DM and T2DM is presented in Figure 1. Autoimmune destruction of islets of Langerhans/*β-*cells leads to the development of T1DM. It has been found that human leukocyte antigen (HLA) encodes cell surface proteins of the islets that interact with immune cells. Activated cytotoxic CD8+ T lymphocytes via the aforementioned HLA genetic factor cause damage to *β-*cells. Furthermore, inflammatory stress caused by cytokines secreted by T lymphocytes also causes damage to *β-*cells. The presence of multiple autoantibodies can be seen in T1DM; however, their contribution to the pathogenesis of T1DM is not confirmed [7]. T2DM occurs due to insulin resistance associated with a defect of compensatory insulin secretion. Insulin resistance is multifactorial and commonly develops with obesity and aging. Genetic factors as well as lifestyle changes affect insulin resistance and then T2DM. Decreased muscle glucose uptake and impaired suppression of hepatic glucose production due to insulin resistance are the main causes of hyperglycemia observed in T2DM. Toxicity exerted by chronic exposure of the *β-*cell to glucose and fatty acids is believed to be leading to *β-*cell damage and dysfunction in T2DM subjects. Chronic hyperglycemia also leads to glycation reactions, and the production of reactive oxygen species causes *β-*cell damage in T2DM [8, 9].

The exact mechanisms of alteration of *β-*cell mass and function in the pathogenesis of DM are still unclear. However, it has been proven that *β-*cell mass and function begin to alter in both T1DM and T2DM even before the symptoms are shown. In T1DM, autoimmune destruction initially reduces *β-*cell mass, and exhaustion of remaining *β-*cells in compensating increased insulin requirement results in subsequent *β-*cell dysfunctions. In T2DM, initially, *β-*cell dysfunction occurs due to exhaustion of *β-*cells in compensating increased insulin requirement imposed by insulin resistance. This leads to a reduction of the *β-*cell mass due to cytotoxicity exerted by hyperglycemic conditions. Hence, in general, therapies targeted at increasing *β-*cell mass and therapies targeted at improving *β-*cell function could be considered as the main treatment and prevention targets of the disease for T1DM and T2DM, respectively [10].

Pancreatic *β-*cell mass and abnormalities of the structure can be assessed by histopathological examination or immunohistochemical analysis of the pancreatic tissue [10, 11]. The histopathological examination involves morphometric analysis such as islet size and density, fractional *β-*cell area, and the number of apoptotic *β-*cells. Immunohistochemical staining using anti-insulin antibodies is important in the assessment of functional *β-*cell mass whereas anti-Ki67 antibodies are used in the assessment of *β-*cell proliferation [12]. Homeostatic model assessment- *β-*cell function (HOMA-*β*) is a mathematical model used for the estimation of *β-*cell function from measurements of fasting plasma glucose and insulin or C-peptide. HOMA-*β* is calculated using the fasting plasma glucose, and insulin hence can be used to evaluate *β-*cell function in the fasting state. For detailed investigation of defects of *β-*cell, dynamic tests such as intravenous glucose, arginine, or glucagon are also used in combination with a hyperglycemic clamp. The *β-*cell function during daily-life conditions is evaluated using an oral glucose tolerance test (OGTT) or a mixed meal tolerance test in combination with an estimation of insulin secretion rates and mathematical modeling [13]. Assessment of gene expressions and protein profiling is also used in assessing *β-*cell regeneration and function. None of the aforementioned methods on its own is considered the gold standard for assessing *β-*cell function. Hence, a combination of several methods is needed for a better conclusion [13].

*β-*Cell regeneration can occur via replication of existing *β-*cell or conversion of other pancreatic cells into *β-*cells. Examples for investigations on experimental drugs derived from the natural origin for their ability to improve *β-*cell regeneration are summarized in Table 1. *β-*Cell replication is mediated by the inhibition of multiple mitogenic signaling pathways. Experimental drugs such as harmine and aminopyrazine compounds have shown *β-*cell regenerative potential in this manner [14, 15]. Modification of distinct genes which are associated with the development of *β-*cell during embryogenesis has a prominent role in converting other cells in the pancreas to *β-*cells. Drugs such as *γ*-aminobutyric acid and artemether act as potential activators of conversion of other cells to *β-*cells [16, 17]. Furthermore, there is a possibility of generating *β-*cells from progenitor cells, including human embryonic and induced pluripotent stem cells derived from somatic cell populations [18].

A *β-*cell function can be restored by stimulating insulin secretory pathways. Sulfonylureas (e.g., glimepiride and glipizide) are believed to stimulate insulin release through activating the K^+^/ATP/sulfonylurea receptor channels. Though these drugs initially reduce hyperglycemia, eventually *β-*cells may get damaged due to exhaustion. Incretin peptides amplify insulin release in a glucose-dependent manner. Glucagon-like peptide 1 (GLP-1) receptor agonists (e.g., polypeptide compounds: liraglutide, exenatide) are effective in promoting biphasic insulin release and rarely cause hypoglycemia or body weight gain. Dipeptidyl peptidase 4 (DPP-4) inhibitors (e.g., vildagliptin and sitagliptin) also contributed to improving *β-*cell function by prolonging the half-life of incretins via prevention of their rapid degradation. It has been suggested that promoting insulin sensitization is a better approach for glycemic control rather than increasing insulin secretion alone which may cause exhaustion of *β-*cells [19].

Available allopathic drugs have not been able to restore *β-*cell function or regeneration and are also associated with side effects such as weight gain, weight loss, hypoglycemia, and abdominal discomforts [20]. Apart from the lifestyle interventions, stem cell treatments and pancreatic islet transplants are also utilized for the management of DM but none has been able to totally manage the disease. Hence, medicinal plants with a history of use in traditional medicine practices and as evident by ethnopharmacological knowledge have gained attention to be used as antidiabetic therapeutics to restore *β-*cell functions and mass due to their multitasking ability as in targeting different aspects of the pathways in *β-*cell regeneration and function [21]. There are limited reviews on these natural products targeting *β-*cells. Oh and Semwal et al. provided comprehensive details on investigations conducted on plant-derived natural products targeting *β-*cells [22, 23]. However, they lack the emphasis on cellular and molecular mechanisms of restoring *β-*cell function and *β-*cell mass, their structure-activity relationships, and toxicity concerns. Ghorbani et al. discussed the cellular and molecular mechanisms of restoring *β-*cell function and *β-*cell mass but the focus was confined to flavonoids [24]. This review attempts to identify the mentioned aspects and elaborate unexplored avenues for future research. We believe that with the given information it would be possible to fill the gap between existing knowledge and unexplored avenues in herbal medicines targeting *β-*cells.

## 2. Description of Study Selection

**Figure 2:**
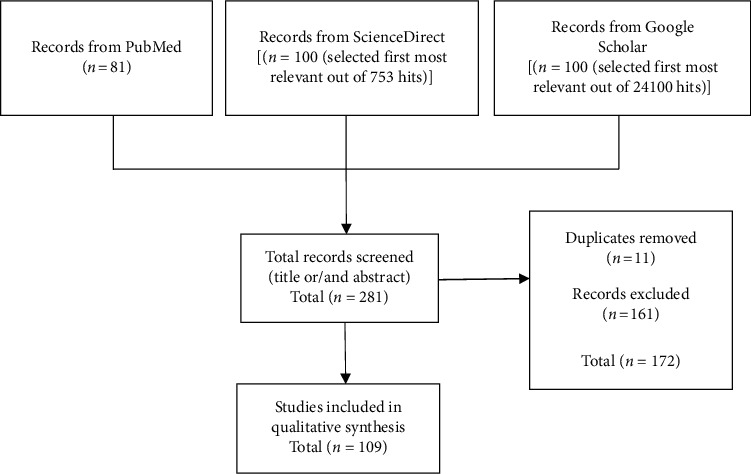
Study selection process.

Online electronic databases “PubMed,” “ScienceDirect,” and “Google Scholar” were searched with the keywords “medicinal plant extracts,” “phytoconstituent,” “*β-*cell regeneration,” “*β-*cell function,” and “diabetes mellitus” in the whole text. A combination of these keywords with relevant Boolean operators was used in searching. Data were collected from January 2009 to March 2020. Studies on the effects of plant crude extracts/fractions/isolated compounds on *β-*cell structure and function using *in vitro* cell culture studies and/or *in vivo* models of T1DM and T2DM were selected for this review. Studies on commercial oral hypoglycemic agents other than plant-derived drugs and synthetic drugs with natural product moiety were excluded. Studies with no direct evidence on *β-*cell function and mass but indirectly suggested improvement of those activities via improving insulin resistance and other antidiabetic parameters were also excluded. Accordingly, in the PubMed database, a total of 81 hits were obtained. Out of them, 39 articles were excluded being not relevant to the scope of this review. The remaining 42 articles were selected for the study. In the ScienceDirect database, under the category of research articles, a total of 753 hits were obtained and 24,100 hits were obtained via Google Scholar. First, the most relevant 100 articles were selected from each database, i.e., ScienceDirect and Google Scholar. Out of 100, 58 articles in ScienceDirect were excluded for being not within the scope, and four articles were excluded being duplications. The remaining 38 articles were selected for the review. Out of 100 articles in Google Scholar, 64 articles were excluded for being not within the scope and seven articles were excluded being duplications. The remaining 29 articles were selected for the review. A total of 109 articles were studied for the review. A summary of the study selection process is shown in Figure 2.

## 3. Results

Plant-derived natural products, i.e., crude plant extracts, fractions, and isolated compounds, have been investigated for antidiabetic activity and are described in the following sections. Among these, some are reported as having potential for improving *β-*cell regeneration and function as evidenced by *in vitro* cell culture studies and animal and human studies. However, only a few natural products have been investigated for detailed mechanisms on regeneration and function of *β-*cell in the above models. *In vitro* cellular models and animal models have been applied widely in elucidating these mechanisms. In many instances, polyphenol compounds including flavonoids that possess pronounced antioxidant activities were found to be improving *β-*cell regeneration and function.

### 3.1. Single Herbal Extracts Targeting *β-*Cells

Extracts of *Artemisia dracunculus* L. (Family: Compositae), *Centaurium erythraea* Rafn (Family: Gentianaceae), *Cornus officinalis* Sieb. et Zucc. (Family: Cornaceae), Gynura divaricata (L.) DC (Family: Asteraceae), *Hibiscus rosa sinensis* Linn. (Family: Malvaceae), *Lactarius deterrimus* (Family: Russulaceae), *Myrica rubra* Sieb. and Zucc. (Family: Myricaceae), *Panax ginseng* C.A. Meyer (Family: Araliaceae), *Tamarindus indica* Linn. (Family: Fabaceae), *Teucrium polium* L. (Family: Lamiaceae), *Thymus praecox* subsp. skorpilii var. skorpilii (Family: Lamiaceae), *Uncaria tomentosa* (Willd.) DC (Family: Rubiaceae), and *Woodfordia fruticosa* (L.) Kurz (Family: Lythraceae) have shown improvement of *β-*cell regeneration and function through different mechanisms. The following sections are focused on the investigations conducted on herbal medicines with the potential for improving regeneration and function of *β-*cell.

#### 3.1.1. *Artemisia dracunculus* L

Ethanol (80% v/v) extract of seeds of *Artemisia dracunculus* was investigated *in vitro* using islets of Langerhans isolated from mouse, NIT-1 cells (a pancreatic *β-*cell line established from a transgenic NOD/Lt mouse), and human islets. Mouse islets and NIT-1 cells were exposed to 1 mM glucose, and human islets were exposed to 2.8 mM glucose prior to treatment with the extract. A significant (*p* < 0.05) increase in glucose-stimulated insulin release was observed in the aforementioned cells when treated with 10 *μ*g/mL of the extract. MTT ((3-(4, 5-dimethylthiazol-2-yl)-2, 5-diphenyltetrazolium bromide)) assay revealed that the extracts are not cytotoxic at tested concentrations. Furthermore, it was revealed that extracts do not increase *β-*cell mass in any of the concentrations. Western blot analysis of protein extract of NIT-1 cells treated with plant extract showed activation of the AMP-activated protein kinase (AMPK) followed by acetyl-CoA carboxylate (ACC) and protein kinase B (PKB/Akt) which was suggested as a possible mechanism for glucose-stimulated insulin secretion [25]. However, the AMPK activity on *β-*cell function and mass is still under debate, and controversial findings are reported among studies conducted to date. There is growing evidence toward the concept that AMPK activation in *β-*cells may cause decreased insulin secretion [26, 27]. There are contradictory theories on the effect of activation of AMPK on *β-*cell apoptosis/survival. However, the activation of Akt has been found to inhibit the AMPK-activated apoptosis of *β-*cells [28]. Hence, the suggested mechanism should be further investigated to ascertain the mechanism of increasing glucose-stimulated insulin secretion and survival of *β-*cells by the investigated plant extract.

#### 3.1.2. *Centaurium erythraea* Rafn

*Centaurium erythraea* is a traditional medicinal plant in Serbia used to treat DM. Methanolic extract of dried aerial parts of *C. erythraea* has been investigated for its mechanisms of improving *β-*cell regeneration and function. *In vivo* studies were carried out using male albino Wistar rats weighing 220–250 g with DM induced by multiple intraperitoneal (i.p.) injections of a low dose of streptozotocin (STZ) (40 mg/kg). Oral administration of the extract at a dose of 100 mg/kg for four weeks resulted in significant improvement of glycemic control as evidenced by significant (*p* < 0.01) reduction of blood glucose level (33%) and increased insulin level (74%) in diabetic rats. Furthermore, hematoxylin and eosin (H&E) stained pancreatic sections showed that the shape, size, and number of islets and the number of *β-*cells returned back to normal upon treatment. Immunohistochemical staining of the pancreatic tissue with anti-insulin antibodies showed an increase in the number and distribution of *β-*cells.

Staining of pancreatic islets for GLUT-2 has shown positive GLUT-2 staining with a uniform distribution of GLUT-2 within the islets. The GLUT-2 is responsible for glucose entry into *β-*cells and thereby stimulating insulin synthesis. Immunohistochemical analysis of the pancreatic tissue of diabetic rats revealed the presence of activated protein kinase B (p-Akt) uniformly distributed throughout the islets. Akt plays an important role in the regulation of pancreatic *β-*cell growth and survival [29]. It has been found that overexpression and activation of Akt in *β-*cells result in an increase in the *β-*cell mass, cell size, and proliferation [30].

Further studies on pancreatic islet tumor Rin-5F cells revealed that cells induced with 12 mM STZ have a significant increase of 12% in cell viability in the presence of 0.25 mg/mL of extract. Furthermore, treatment with the extract showed a 77% increase in insulin 1 (Ins1) gene (i.e., the gene encoding insulin) expression and a 90% increase in insulin secretion and protection of DNA from damage. Treatment with the extract also exhibited an antioxidant effect on *β-*cells in terms of reduction of lipid peroxidation [malondialdehyde (MDA) level (27%), protein *S*-glutathionylation (GSSP) (40%)] and increased ratio of reduced glutathione/oxidized glutathione (GSH/GSSG) (8%). Amelioration of STZ-induced oxidative stress was evident by decreased activity of antioxidant enzymes including manganese superoxide dismutase (MnSOD) (22%), copper-zinc superoxide dismutase (CuZnSOD) (11%), and catalase (CAT) (to a normal level). Furthermore, it was observed that STZ-induced oxidative stress caused imbalances in activities of redox-sensitive transcription factors, nuclear factor-*κ*B-p-65 subunit (NF-*κ*B-p65), nuclear factor erythroid 2-related factor 2 (Nrf-2), and specificity protein 1 (Sp1) which are involved in the transcriptional regulation of aforementioned antioxidant enzymes. Treatment with the extract resulted in the readjustment of these factors by reducing NF-*κ*B-p-65 (24%) and Nrf-2 (3-fold) and increasing Sp1 (to a normal level) [29]. Furthermore, it was revealed that the extract contains secoiridoids (secologanin, sweroside, swertiamarin, gentiopicrin, and loganin) and polyphenols including phenolic acids (sinapic acid, caffeic acid, ferulic acid, and *p*-coumaric acid), flavonoids, i.e., flavones (apigenin and luteolin), flavonols (rutin, isoquercitrin, astragalin, kaempferol, quercetin), flavanones (naringenin), and xanthones (eustomin, desmethyleustomin, methylbellidifolin, and decussatin) [31].

#### 3.1.3. *Cornus officinalis* Sieb. et Zucc

Aqueous extract of dried fruits of *Cornus officinalis* at a dose of 500 *μ*g/mL was investigated in a T1DM model using human 1.1B4 pancreatic *β-*cells. The extract accelerated *β-*cell regeneration following cytokine-induced death. Furthermore, the treatment showed a 2-fold increase in cell proliferation. The plant extract was found to be rich in iridoid glycosides. It was suggested that the effects might be due to the observed twofold increase in the gene expression of the calcium-dependent transcription factor, nuclear factor of activated T cells, and cytoplasmic 2 (NFATC2) [32]. NFATC2 is considered the key transcriptional factor which regulates transcription of many genes associated with DM thereby promoting *β-*cell proliferation and function [33].

#### 3.1.4. *Gynura divaricata* (L.) DC. Gynura divaricata

 is a traditional Chinese medicinal herb with antidiabetic activity. Methanolic extract of aerial parts of *G. divaricata* has been investigated in ICR mice (three weeks of age) with T2DM induced by a high-fat diet followed by i.p. injection of STZ (100 mg/kg). It was found that the crude extract contains polyphenolic compounds including 3-caffeoylquinic acid, 4,5-dicaffeoylquinic acid, 3,4-dicaffeoylquinic acid, and 3,5-dicaffeoylquinic acid. The incorporation of 10% *G. divaricata* to the diet of the diabetic mice for four weeks caused a marked recovery of pancreatic islets with an increase of *β-*cell counts, and an improvement of islet structure which was observed on H&E staining. An immunohistochemical study using anti-insulin antibodies showed an increased number of insulin-positive *β-*cells reflecting *β-*cell regeneration. A significant (*p* < 0.01) increase in the expression of GLUT-2, glucokinase (GK), v-maf musculoaponeurotic fibrosarcoma oncogene family protein A (MafA), and pancreatic duodenal homeobox-1 (PDX-1) was observed reflecting increased functioning of pancreatic *β-*cells. PDX-1 and MafA are important transcription factors which regulate gene expressions in *β-*cells. PDX-1 is essential for the development of pancreatic cells including *β-*cells and involves insulin gene transcription. MafA binds to the promoter region of the insulin gene promoting insulin expression in response to glucose [34]. An increase in the expression of antiapoptotic protein and B-cell lymphoma-2 (Bcl-2) and a decrease in the expression of apoptotic proteins, Bcl2-associated X (Bax), and cysteinyl aspartate specific proteinase-3 (caspase-3) suggesting *β-*cell survival were also observed. Accordingly, *G. divaricata* crude extract showed pronounced hypoglycemic effects by inhibiting islet cell apoptosis and improving pancreatic function [35].

#### 3.1.5. *Hibiscus rosa sinensis* Linn

**Figure 3:**
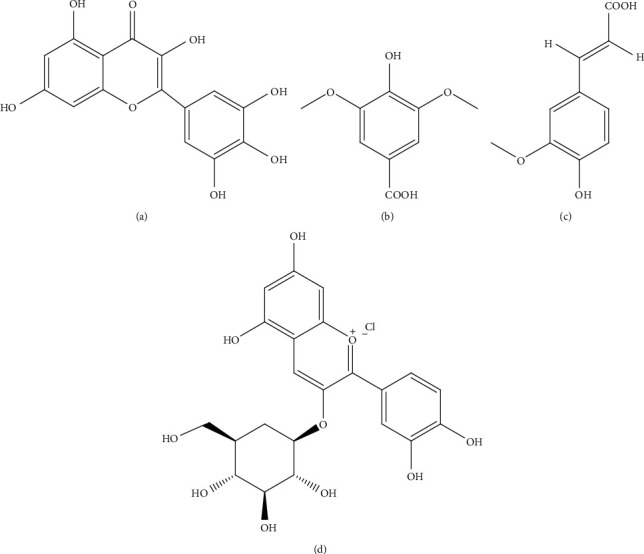
Structures of metabolites: (a) myricetin (**1**), (b) syringic acid (**2**), (c) ferulic acid (**3**) present in *Hibiscus rosa sinensis* Linn., and (d) cyanidin-3-glucoside (**4**) present in *Myrica rubra* Sieb. and Zucc. targeting *β-*cells.

*Hibiscus rosa sinensis* is an Ayurvedic medicinal plant used in India for the treatment of DM. Aqueous extract of flower petals of *H. rosa sinensis* was investigated in glucose-stressed RIN-m5F cells. Cells treated with 50 *μ*g/mL extract elevated the release of insulin and modulated apoptotic signaling pathways in *β-*cells. It significantly (*p* < 0.05) reduced NF-*κ*B nuclear translocation, thereby downregulated the expressions of major inflammatory cytokines, and upregulated expressions of pancreatic *β-*cell functional genes, i.e., forkhead box O1 (FOXO-1), Urocortin 3 (Ucn-3), PDX-1, MafA, and natural killer cell transcription factor‐related, gene family 6, locus 1 (Nkx6.1) suggesting its protective effects on *β-*cells. Major constituents of the extract responsible for these activities are supposed to be polyphenols including myricetin (**1**), syringic acid (**2**), and ferulic acid (**3**) (Figure 3) [36].

#### 3.1.6. *Lactarius deterrimus*

Ethanol (50% v/v) extract of dried mushroom *Lactarius deterrimus* was investigated in 2.5- month-old adult albino Wistar rats weighing 220–250 g with DM induced by multiple low dose STZ (40 mg/kg/day) i.p. injection. Intraperitoneal administration of the extract (60 mg/kg) daily for four weeks increased the expression of chemokine CXCL12 protein. CXCL12 protein is involved in the activation of the serine/threonine-specific Akt prosurvival pathway causing restoration of *β-*cell population. *β-*Cell regeneration was further emphasized by an increase in the numbers of proliferating cell nuclear antigen (PCNA), i.e., an intranuclear polypeptide expressed during cell proliferation, and insulin-positive *β-*cells. These results indicated the potential of the *L. deterrimus* extract to alleviate oxidative stress and increase *β-*cell mass [37].

#### 3.1.7. *Myrica rubra* Sieb. and Zucc

*Myrica rubra* (bayberry) fruit extract rich in cyanidin-3-glucoside (**4**) (Figure 3) was investigated *in vitro* using pancreatic *β-*cells and *in vivo* using mice induced with DM by i.p. injection of STZ (200 mg/kg). Pretreatment of *β-*cells exposed to hydrogen peroxide (H_2_O_2_) with the extract at a dose of 15 *μ*g/g resulted in increased cellular viability and decreased mitochondrial reactive oxygen species production and prevented cell death and cell necrosis. Furthermore, the extract upregulated PDX-1 gene expression, contributing to increased gene transcription of insulin-like growth factor 2 (IGF-2) and insulin in INS-1 cells (rat insulinoma cell line, a *β-*cell line) [38].

#### 3.1.8. *Panax ginseng* C.A. Meyer

Butanol extract of *Panax ginseng* was investigated in mice with DM induced by i.p. injection of STZ at a dose of 80 mg/kg and INS-1 cells. The oral administration of the extract (200 mg/kg) for diabetic rats for 10 weeks resulted in enhanced glucose-stimulated insulin secretion and *β-*cell proliferation. Incubation of INS-1 cells with the extract for 24 hours resulted in an increase in the number of *β-*cells. Furthermore, the treatment of INS-1 cells with the extract (5 *μ*g/mL) increased the gene expressions of cyclin D2, PDX-1, and insulin receptor substrate 2 (IRS-2) which were suggested as the mechanism of *β-*cell proliferation. Ginsenosides (steroid glycosides) present in the extract would be causing the observed biological effects [39].

#### 3.1.9. *Tamarindus indica* Linn

Aqueous extract of seeds of *Tamarindus indica* was investigated in rats induced with DM by i.p. injection of STZ (90 mg/kg). Oral administration of 240 mg/kg extract for four weeks resulted in a slight decrease in the number of insulin-positive granules compared to the control group. Furthermore, islet atrophy was not detected compared to the control group indicating protective effects against *β-*cell damage. The anti-inflammatory activity of the extract was represented by a significant (*p* < 0.05) reduction of tumor necrosis factor-*α* (TNF-*α*) level. An increased insulin secretion via increasing (*p* < 0.001) cytosolic Ca^2+^ levels was observed in islets isolated from rats upon treatment with 100 *μ*g/mL extract [40].

#### 3.1.10. *Teucrium polium* L

Hydroalcoholic extract of aerial parts of *Teucrium polium* was investigated for its antidiabetic activity in rats induced with DM by single i.p. injection of STZ (45 mg/kg). The administration of 0.5 g/kg extract to diabetic rats for 42 days resulted in increased *β-*cell mass. Treatment with *T. polium* extract also caused a reduction in the MDA (64%) level and increment of the pancreatic superoxide dismutase (SOD) (EC 1.15.1.1) (45%), CAT (EC 1.11.1.6) (52%), and GSH (105%) contents indicating amelioration of oxidative stress. Assessment of transcription factors showed higher levels of PDX-1 in diabetic rats while the level of FOXO-1 decreased. c-Jun N-terminal kinase (JNK) activation under oxidative stress, which leads to FOXO-1 formation, is a molecular mechanism related to the destruction of *β-*cells. Nuclear accumulation of FOXO-1 is believed to reduce the PDX-1 expression thereby reducing insulin gene expression. PDX-1 is also important in the restoration and proper function of the insulin-producing *β-*cells [41].

#### 3.1.11. *Thymus praecox* subsp. skorpilii var. skorpilii

*Thymus praecox* is consumed as a decoction in Turkish folk medicine for the treatment of diabetes. Methanolic extract of aerial parts of this plant was investigated on STZ (55 mg/kg)/nicotinamide (NA) (100 mg/kg)-induced type 2 diabetic rats. Oral administration of the plant extract at a dose of 100 mg/kg for three weeks resulted in significant (*p* < 0.01) reduction of the proinflammatory cytokines (TNF-*α*, interleukin-1*β-*(IL-1*β*), interleukin-6 (IL-6)), and significant (*p* < 0.001) elevation of glucagon-like peptide-1 (GLP-1) in the pancreas. Histopathological assessment of the pancreas showed protection of *β-*cells from STZ/NA-induced damage [42].

#### 3.1.12. *Uncaria tomentosa* (Willd.) DC

*Uncaria tomentosa* is a native species to the Amazon rain forest and is widely used as traditional medicine for DM. Ethanol (50% v/v) extract of stem bark of *U. tomentosa* was investigated in six-week-old C57BL/6 male mice with DM induced by five i.p. injections of STZ (40 mg/kg). Oral administration of *U. tomentosa* extract (400 mg/kg) for 21 days showed a higher number of intact islets and a significant inhibition of destructive insulitis in histopathological examination of the pancreatic tissue. Furthermore, the immunohistochemical evaluation showed an increase of insulin-positive *β-*cells. The phenotypic analysis indicated that treatment with higher doses (100–400 mg/kg) resulted in CD4(+) and CD8(+) T-cell values similar to healthy animals. Same doses also increased the number of CD4(+), CD25(+), and Foxp3(+) regulatory T cells. Furthermore, the extract modulated the production of Th1 and Th2, with increased levels of IL-4 and IL-5. Altogether, results suggest the immunomodulatory action of extract and protection of *β-*cells from immune reactions.

**Figure 4:**
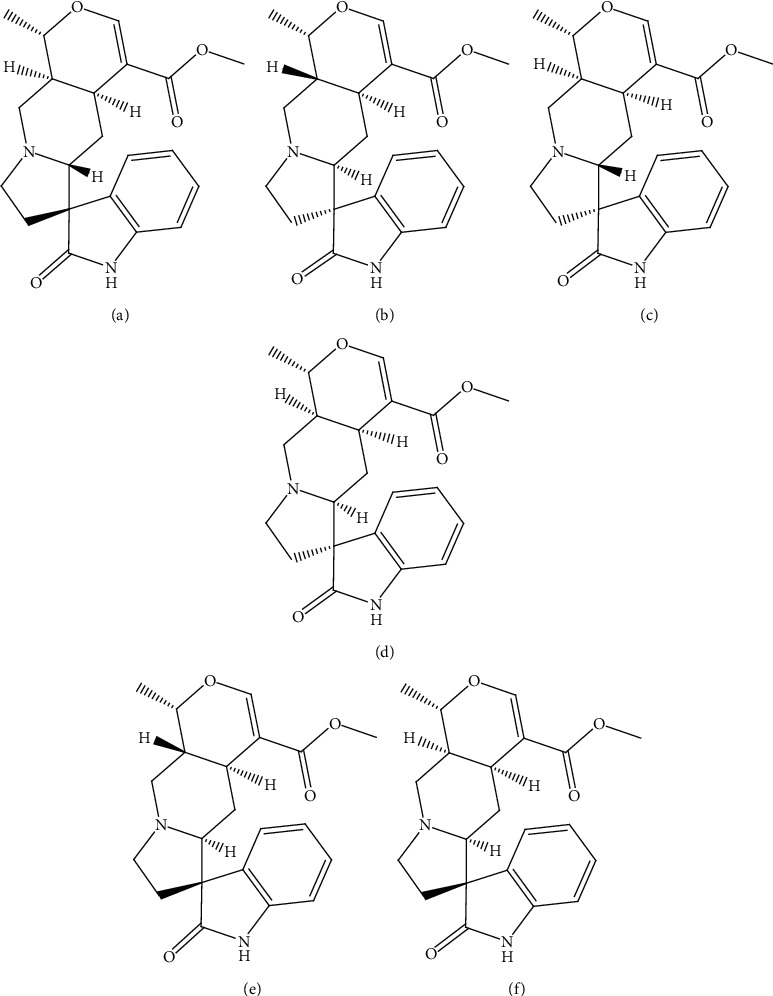
Structures of metabolites (a) speciophylline (**5**), (b) mitraphylline (**6**), (c) uncarine F (**7**), (d) pteropodine (**8**), (e) isomitraphylline (**9**), and (f) isopteropodine (**10**) present in *Uncaria tomentosa* (Willd.) DC targeting *β-*cells.

Pentacyclic oxindole alkaloids, i.e., speciophylline (**5**), mitraphylline (**6**), uncarine F (**7**), pteropodine (**8**), *iso*-mitraphylline (**9**), and *iso*-pteropodine (**10**) (Figure 4), present in the extract exert the reported therapeutic activities [43].

#### 3.1.13. *Woodfordia fruticosa* (L.) Kurz

Methanolic extract of flowers of *Woodfordia fruticosa* was investigated in Sprague–Dawley rats with diabetes induced by STZ (45 mg/kg)–nicotinamide (120 mg/kg) i.p. injection. Treatment of diabetic rats with extract (200 mg/kg) for 45 days ameliorates oxidative stress by significantly (*p* < 0.05) downregulating lipid peroxidation levels. Furthermore, the histopathological analysis showed recovery in the structural degeneration of *β-*cell mass. The immunohistochemical study showed upregulation in insulin. Western blot analysis showed a slight increase in the GLUT-2 protein expression in the pancreas [44].

### 3.2. Compounds Isolated from Plants Targeting *β-*Cells

#### 3.2.1. Salidroside

**Figure 5:**
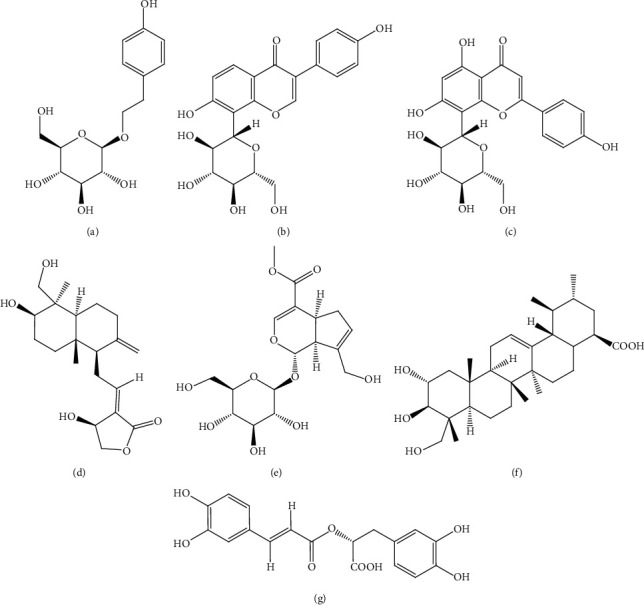
Structures of metabolites (a) salidroside (**11**), (b) puerarin (**12**), (c) vitexin (**13**), (d) andrographolide (**14**), (e) geniposide (**15**), (f) asiatic acid (**16**), and (g) rosmarinic acid (**17**) isolated from medicinal plants targeting *β-*cells.

Salidroside (**11**) (Figure 5) is a phenylethanoid glycoside extracted from the medicinal plant *Rhodiola rosea* L. (Family: Crassulaceae). This compound was investigated in four-week-old male C57BL/6 mice fed with a high-fat diet. Oral administration of salidroside (100 mg/kg/day) for five weeks increased *β-*cell mass and *β-*cell replication of diabetic mice. The effects of salidroside under diabetic stimuli (glucose/cytokines) were further investigated in Min6 cells (a pancreatic *β-*cell line). Under diabetic stimuli, salidroside suppressed reactive oxygen species production and restored mitochondrial membrane potential via reducing NADPH oxidase 2 (NOX2) expression and inhibiting JNK-caspase 3 apoptotic cascade subsequently leading to *β-*cell survival. Simultaneously, it reversed diabetes-associated oxidative stress by activating AMPK-Akt to inhibit FOXO1 and recovering PDX-1 nuclear localization. Results indicated that salidroside prevents *β-*cell failure via AMPK activation [45].

#### 3.2.2. Ginseng Oligopeptides

*Panax ginseng* Meyer (Family: Araliaceae) is a traditional Chinese medicinal herb used to treat DM. Administration of ginseng oligopeptides (0.5 g/kg) for 52 days to Sprague–Dawley rats (5 weeks old, 130–170 g) with DM induced by high carbohydrate/high-fat diet followed by alloxan (105 mg/kg) injection resulted in an increase (*p* < 0.05) in the HOMA-*β* values. The increase in insulin level and the decrease in expression of NF-*κ*B and Bcl-2 family in pancreatic islets were also detected by Western blot analysis. Histopathological examination of the pancreas of treated animals showed amelioration of pancreatic damage. Furthermore, the *β-*cell survival time and rate were significantly longer in treated animals [46].

#### 3.2.3. Puerarin

Puerarin (**12**) (Figure 5), an isoflavone from the root of *Pueraria lobata* (Willd.) Ohwi. (Family: Fabaceae), was investigated *in vivo* for its *β-*cell regeneration potential via glucagon-like peptide 1 receptor (GLP-1R) signaling activation. Oral administration of puerarin (150 mg/kg) daily for 10 days to C57BL/6 diabetic mice fed with a high-fat diet increased the HOMA-*β* and *β-*cell proliferation in pancreatic sections. The expression of transcription factors PDX-1 and neurogenin 3 (Ngn3) and upregulation of GLP-1R signaling in isolated pancreatic ductal epithelial cells indicated *β-*cell neogenesis. Small islet-like cell clusters (ICCs) were observed indicating ductal epithelium differentiation. Efficacy of puerarin was suppressed *in vivo* by GLP-1R antagonist exendin-9-39 but was enhanced by exendin-4, a GLP-1R agonist indicating the effect of puerarin in GLP-1R activation [47].

#### 3.2.4. Vitexin

Vitexin (**13**) (Figure 5) is a *C*-glycosyl flavonoid isolated from leaves of *Ficus deltoidea* Jack (Family: Moraceae). INS-1 cells pretreated with vitexin (20 and 40 *μ*M) followed by exposure to high glucose (33 mM) were protected against cytotoxicity exert by high glucose concentration. Vitexin improved insulin signaling as analyzed by the levels of functional proteins including GLUT-2, insulin receptor (IR), IRS-1, and IRS-2 and causing glucose-stimulated insulin secretion. Vitexin improved the high glucose-induced nuclear transcription factor system by suppressing Rel A, Rel B, I*κ*B, and P50/p105 expression resulting in decreased cell apoptosis, further confirmed by the reduction in the percentage of Annexin-V (i.e., a fluorescent stain for detect apoptotic cells) positive cells. Furthermore, vitexin activated proteins including NF-*κ*B and Nrf2 in *β-*cells regulating apoptosis [48].

#### 3.2.5. Saponins

Saponins isolated from *Momordica charantia* L. (Family: Cucurbitaceae) on insulin secretion were investigated on INS-1 pancreatic *β-*cells exposed to high glucose concentration (33.3 mM). Treating these cells with saponins caused improved cell morphology and increased insulin secretion. The treatment also increased the expression of IRS-2 and increased the phosphorylation of Akt protein and decreased the protein level of FOXO-1. Furthermore, saponins increased the level of PDX-1. Saponins also increased glucose-stimulated insulin secretion and intracellular insulin content. All these expressions were reversed by phosphoinositide 3-kinase (PI3K) indicating that saponins may increase insulin secretion via the phosphatidylinositol 3-kinase (PI3K)/Akt/FOXO-1 signaling pathway [49].

#### 3.2.6. Andrographolide-Lipoic Acid Conjugate

Andrographolide (**14**) (Figure 5) is a diterpenoid isolated from *Andrographis paniculata* (Burm. f.) Wall. ex Nees. (Family: Acanthaceae). Conjugation of andrographolide with natural antioxidant lipoic acid at 80 mg/kg dose was investigated in BALB/c mice induced with T1DM by intravenous injection of alloxan (60 mg/kg) and in RIN-m cell line (an insulinoma cell line). Treatment with the aforementioned compound for six days resulted in increased *β-*cell mass in treated mice. Immunohistochemical staining using an anti-insulin antibody showed increased insulin-containing *β-*cells. Pretreatment of the H_2_O_2_-treated RIN-m cells with conjugate (1 *μ*M) increased the cell viability to 62.2% and reduced the reactive oxygen species level indicating protection of cells from oxidative stress. At 1 *μ*M concentration, this conjugate completely blocked IL-1*β-*and interferon-*γ* (IFN-*γ*) induced NF-*κ*B activation thereby protecting *β-*cells from apoptosis. [50].

#### 3.2.7. Geniposide

Geniposide (**15**) (Figure 5), an iridoid glycoside isolated from *Gardenia jasminoides* Ellis (Family: Rubiaceae), at a dose of 100 mg/kg given for 56 days was found to be causing *β-*cell regeneration in investigations conducted using C57BL/6J mice with DM induced by feeding high-fat diet. Further investigations using Min6 cells revealed that geniposide (20 *μ*M) resulted in increased expression of T-cell factor 7-like 2 (TCF7L2), a transcription factor of Wnt/*β*-catenin signaling which involves in *β-*cell survival and regeneration. Geniposide promotes *β-*cell survival by increasing proliferation of *β-*cell and decreasing *β-*cell apoptosis in cultured mouse islets after being challenged with diabetic stimuli (glucose/inflammatory cytokines). The compound further activated Akt, enhanced the expressions of GLP-1R inhibited GSK3*β-*activity, and promoted *β*-catenin nuclear translocation. Geniposide induced small islet-like cell clusters formation as a result of *β-*cell neogenesis from ductal epithelium [51].

#### 3.2.8. Asiatic Acid

Asiatic acid (**16**) (Figure 5) is a triterpenoid found in plants including *Centella asiatica* (L.) Urb. (Family: Apiaceae). Wistar rats with DM induced by i.p. injection of STZ (60 mg/kg) were treated with asiatic acid (25 mg/kg) for two weeks. Treatment caused improved Akt and Bcl‐xL expression in the pancreatic islets of rats as evaluated by Western blot methods. Immunohistochemical staining revealed preservation of insulin-producing *β-*cells in the pancreatic islets of diabetic rats. Furthermore, asiatic acid resulted in prosurvival Akt kinase activation and BclxL expression in the pancreatic islets of diabetic rats [52].

#### 3.2.9. *Angelica sinensis* Polysaccharide (ASP)

ASP derived from the roots of *Angelica sinensis* (Oliv.) Diels (Family: Apiaceae) was investigated on pancreatic islets of BALB/c mice with T2DM induced by feeding a high-fat diet followed by i.p. injection of STZ (60 mg/kg). Treatment with ASP (100 mg/kg) for eight weeks showed antiapoptotic function on *β-*cells via increasing the Bcl-2/Bax ratio thereby blocking caspase-9-caspase-3 cascade. Simultaneous suppression of caspase-8-driven extrinsic apoptotic pathway further inhibited the activation of proteins such as poly (ADP-ribose) polymerase (PARP) involved in *β-*cell destruction. Immunohistochemistry and immunocytochemistry indicated that ASP could increase intracellular insulin. ASP also promoted the secretion of insulin by stimulating the insulin gene expression [53].

#### 3.2.10. Phenylpropenoic Acid Glucoside (PPAG)

Treatment with PPAG isolated from *Aspalathus linearis* (Burm.f.) R.Dahlgren (Family: Fabaceae) using obese mice fed with a high-fat diet in conjunction with fructose caused tripling of *β-*cell mass. Mechanism of the increase of *β-*cell mass was investigated by Ki67 immunostaining (an indicator of *β-*cell proliferation) and by genetic lineage tracing (an indicator of *β-*cell neogenesis) of pancreatic tissue, and both were not detected in this case. In contrast, terminal deoxynucleotidyl transferase dUTP nick end labeling (TUNEL) staining which is used to detect DNA breaks formed during the final phase of cell apoptosis revealed suppressed apoptosis in PPAG-treated obese mice. *In vitro* studies conducted using isolated *β-*cells from mice and INS-1E cell lines showed that PPAG protected *β-*cells from palmitate-induced (i.e., a model of lipotoxicity) apoptosis. Exposure of PPAG-treated cells to different stresses revealed that PPAG protected *β-*cells against endoplasmic reticulum (ER) stress. Further Western blot analysis revealed that this protection is conferred by increasing the expression of antiapoptotic Bcl2 protein in *β-*cells without affecting proapoptotic signals [54].

In another study, PPAG has investigated *in vivo* using BALB/c mice induced with DM by i.p. injection of STZ (200 mg/kg) and *in vitro* using INS-1E *β-*cells and human pancreatic islet cells. Treatment with the PPAG (10 mg/kg) protected *β-*cells from apoptotic cell death and prevented loss of expression of antiapoptotic protein Bcl2. *In vitro* studies showed that PPAG protected INS-1E *β-*cells from STZ-induced apoptosis in a Bcl2-dependent and independent way, respectively, depending on glucose concentration. PPAG also protected human pancreatic islets against the cytotoxic action of palmitate [55].

### 3.3. Polyherbal Mixtures with Potential for *β-*Cell Regeneration and Function

*Sangguayin* is a traditional Chinese medicine prepared using the leaf of *Morus alba* L. (Family: Moraceae), the root of *Pueraria lobata* (Willd.) Ohwi (Family: Fabaceae), dried rhizoma of *Dioscorea opposita* Thunb. (Family: Dioscoreaceae), and fruits of *Momordica charantia* L. (Family: Cucurbitaceae). Sangguayin was investigated using diabetic mice and mouse insulinoma 6 (MIN6) cells for its antiapoptotic action. Treatment with sangguayin (175 mg/kg) for nine weeks reduced pancreatic pathological changes and islet *β-*cell apoptosis in db/db diabetic mice. Pretreatment with sangguayin (25–100 *μ*g/ml) resulted in decreased palmitate-induced apoptosis in MIN6 cells. Decreased expression of cleaved PARP, cleaved caspase-3, and Bax, and increased Bcl-2 reflected *β-*cell survival upon treatment. Furthermore, the treatment suppressed ER stress which was reflected by decreased ER stress pathway-related proteins (Bip/XBP1/IRE1*α*/CHOP/Caspase-12) and autophagy as indicated by downregulation of autophagy-related protein expressions (LC3/p62/Atg5) [56].

Several other polyherbal mixtures were found in the literature as having potential for improving *β-*cell regeneration and function. However, the mechanisms of their actions are not yet investigated.

A polyherbal mixture comprised of aqueous extracts of leaves of *Murraya koenigii* L. (Family: Rutaceae), cloves of *Allium sativum* L. (Family: Amaryllidaceae), fruits of *Garcinia quaesita* Pierre (Family: Clusiaceae), and seeds of *Piper nigrum* L. (Family: Piperaceae) was investigated in Wistar rats with DM induced by i.p. injection of STZ (70 mg/kg). Oral administration of extract in a dose of 1 g/kg for 30 days resulted in improvement of HOMA-*β*. Further regeneration of pancreatic *β-*cells was observed in histopathological studies [57].

A polyherbal mixture consisting of 95% ethanol extract of seeds of *Nigella sativa* L. (Family: Ranunculaceae) and bark of *Cinnamomum cassia* (L.) D. Don (Family: Lauraceae) was investigated in Wistar rats with DM induced by i.p. injection of STZ (45 mg/kg). Oral administration of the extract at a dose of 100–200 mg/kg for 30 days resulted in reversal of STZ-induced pancreatic cell damage as observed from histopathological studies [58].

A polyherbal mixture comprised of 80% ethanol extract of leaves of *Vernonia amygdalina* Del. (Family: Asteraceae) and *Gongronema latifolium* (Utazi) (Family: Apocynaceae) was investigated in albino rats with DM induced by i.p. injection of STZ (65 mg/kg). Oral administration of the extract at a dose of 200 mg/kg for 28 days resulted in regeneration of pancreatic *β-*cells as evident by histopathological studies [59].

### 3.4. Clinical Studies on Herbal Medicines with Potential for *β-*Cell Regeneration and Function

Only a few clinical studies were reported highlighting the potential for *β-*cell regeneration and function by herbal medicines. However, the detailed mechanisms of action of these medicines are yet to be investigated.

Diabetes tea™, a black tea originating from *Camellia sinensis* (L.) Kuntze var. sinensis (Family: Theaceae) which is supplemented with 12 other medicinal plants, has been investigated in patients with T2DM in a double-blind clinical trial. Administration of 2.5 g of tea powder for 12 weeks resulted in a significant (*p* < 0.05) decrease in HbA1C and LDL. Furthermore, the treatment caused suppression of CD4+ T-cell expression of IL-1*β-*and IL-8 and upregulation of the expression of IL-10 and the Treg/IL-17 ratio. Suppression of these proinflammatory cytokines is beneficial for the protection of *β-*cells from cytokine-mediated damage [60].

A 70% ethanol extract of aerial parts of *Melissa officinalis* L. (Family: Lamiaceae) was investigated in patients with T2DM in a double-blind clinical trial. Rosmarinic acid (**17**) (Figure 5) was found to be the main component of this extract. Administration of a capsule containing 700 mg of extract for 12 weeks resulted in improvement of *β-*cell activity as evident by significant (*p* < 0.05) improvement of HOMA- *β-*[61].

A crude drug combination used in traditional Chinese medicine has been investigated in patients with T2DM in a double-blind clinical trial. The crude drug combination consists of *Coptis chinensis-*rhizome (50%) and Shen-Ling-Bai-Zhu-San (SLBZS) (50%). SLBZS consists of *Panax ginseng* C.A.Mey.-root, *Poria cocos* (Schw.) Wolf-inner parts of the sclerotia, *Atractylodes macrocephala* Koidz.-rhizome, *Dolichos lablab* L.-seed, *Dioscorea opposita* Thunb.-rhizome, *Nelumbo nucifera* Gaertn.-seed, *Platycodon grandiflorus* (Jacq.) A.DC.-root, *Coix lacryma*-jobi L. var. ma-yuen (Roman.) Stapf-seed, *Amomum villosum* Lour.-fruit, *Ziziphus jujuba* Mill.-fruit, and *Glycyrrhiza uralensis* Fisch.-root and rhizome at a 3 : 3 : 3 : 2.3 : 3 : 1.5 : 1.5 : 1.5 : 1.5 : 1.5 : 3 ratio. Oral administration of 9 g of the above-mentioned combination daily for 12 weeks resulted in increased pancreatic *β-*cell function as evident by increased HOMA-*β-*value by 68.9% [62].

Several other plant extracts and isolated compounds exerted antidiabetic effects via targeting *β-*cells. However, the mechanisms have not yet been investigated in depth. These are included in Tables 2 and 3, respectively.

## 4. Discussion

A study of 109 full articles published during the last 10 years led to the identification of single herbal extracts, isolated compounds, and polyherbal mixtures derived from approximately 110 medicinal plants belonging to 51 plant families having potential for improving pancreatic *β-*cell function and regeneration. The most commonly reported plant families include Fabaceae, Cucurbitaceae, Apocynaceae, Lamiaceae, and Rosaceae. The majority of the investigations were conducted as *in vitro* and/or preclinical studies. Only 2.8% of investigations were clinical trials. Hence, there are many unexplored avenues in the topic of this review where future research should be oriented in order to develop safe and effective novel pharmaceuticals/nutraceuticals against diabetes mellitus.

Herbal medicines identified in this review exerted their effects on *β-*cells via different mechanisms which are presented in Figure 8. Many herbal medicines reported improvement of *β-*cell function as observed through HOMA-*β-*value (an index of insulin secretory function) derived from fasting plasma glucose and insulin concentrations. Pancreatic *β-*cell regeneration was observed in histopathological studies via increase of size and number of pancreatic islets and in immunohistochemical studies via semiquantitative and quantitative estimations of insulin-positive cells and proliferating *β-*cells. Western blot analysis and immunohistochemistry to identify transcriptional factors related to gene expressions and other proteins in *β-*cell were found to be commonly used in elucidating mechanisms of action of herbal medicines identified in this review. Increasing *β-*cell mass via the expression of genes/proteins related to antiapoptotic actions (e.g., *Angelica sinensis* polysaccharide, vitexin, aqueous extract of *Hibiscus rosa sinensis*, methanolic extract of *Woodfordia fruticosa*, salidroside, and sangguayin) and *β-*cell neogenesis (e.g., geniposide) and proliferation (e.g., aqueous extract of *Cornus officinalis*, PPAG) identified as mechanisms of *β-*cell regeneration in some herbal products. Apoptosis could occur when the proapoptotic Bcl-2 proteins (Bad, Bid, Bik, and Bax) exceed antiapoptotic proteins of the Bcl family (Bcl-2 and Bcl-xL) in the mitochondrial membrane [139]. Increased expression of antiapoptotic proteins with decreased expression of proapoptotic proteins was observed in herbal medicines with antiapoptotic action. Increasing glucose-stimulated insulin secretion via activating GLUT-2 receptors (e.g., methanolic extract of *Centaurium erythraea*) and increasing intracellular Ca^2+^ levels (e.g., aqueous extract of *Tamarindus indica*) were also observed upon treatment with some herbal medicines. Most of the studies suggested that reduction of oxidative stress by natural compounds with antioxidant potential protects *β-*cells from damage from reactive oxygen species. Herbal medicines with the ability to reduce proinflammatory cytokines also exerted protective effects on *β-*cells. Furthermore, the herbal medicines act on various insulin signaling pathways including activation of PIK3/Akt/FOXO1 pathways, increasing expression of transcriptional factors AMPK, PDX1, and NFkB, and activation of GLP-1R. However, there are knowledge gaps and unexplored avenues on mechanisms of action of herbal medicines on pancreatic *β-*cells which should be further investigated in order to develop effective therapy against DM.

Though the exact cellular and molecular mechanisms of *β-*cells causing pathogenesis of diabetes mellitus are still under investigation, certain therapeutic targets in *β-*cell for the bioactive compounds have already identified such as nuclear receptors (e.g., peroxisome proliferator-activated receptors (PPARs)), cell membrane receptors (e.g., K^+^/ATP channels, Na^+^/Ca^2+^ exchanger, GLP-1R), transcription factors (e.g., PDX1, Ngn3), and intracellular enzymes (e.g., glucokinase) [140, 141]. Bioactive compounds specifically acting on the aforementioned therapeutic targets could be an effective approach in developing therapies against DM. However, a limited number of publications are available on the structure-activity relationship (SAR) of natural products targeting *β-*cells; hence, this aspect needs to be addressed.

It was observed that the majority of the natural bioactive compounds responsible for *β-*cell regeneration and improvement of *β-*cell function are phenolic compounds including polyphenols such as flavonoids. The SAR of polyphenols as antidiabetic agents is still unclear. However, the occurrence of multiple-OH groups attached to the aromatic ring and the arrangement of these hydroxyls in the ortho-dihydroxy conformation are supposed to be significant structural characteristics for high radical scavenging activity [142]. Most of the polyphenols share those characteristics and hence possess radical scavenging activity. However, the pharmacophore related to antioxidant activity may differ among compounds and hence should be investigated for each compound separately [143]. The amphiphilic nature of most of the polyphenols allows interactions with membrane components at different levels thereby generating cellular responses [144]. In the case of flavonoids, the total number and the configuration of hydroxyl groups are found to be important in regulating antioxidant and antidiabetic properties. C-2-C-3 double bond and C-4 ketonic group are two essential structural features for the antidiabetic property of flavonoids [145].

A limited number of studies have been conducted on SAR of isolated compounds found in this review. Salidroside (**11**) (Figure 5) is a phenylethanoid glycoside of which the aglycone is a tyrosol. Upon *in vivo* administration, salidroside gets metabolized into *p*-tyrosol (**31**) (Figure 9) which accounts for the bioactivity of salidroside [146]. Small peptides (molecular weights of less than 1 kDa) such as *Panax ginseng* oligopeptides showed higher antioxidant capacity and strong immunomodulatory action. However, the SAR of oligopeptides is still unclear [147]. Free hydroxyl groups on the B ring are found to be responsible for the antioxidant properties of *C*-glycosylflavones such as vitexin (**13**) (Figure 5) [148]. *C*-glycosides such as vitexin and *O*-glycosides such as salidroside (**11**) exert similar biological effects. However, both vitexin and *O*-glycosides have poor water solubility. Hence, modification of glycosidic bonds to improve water solubility would lead to improved bioavailability [149].

**Figure 6:**
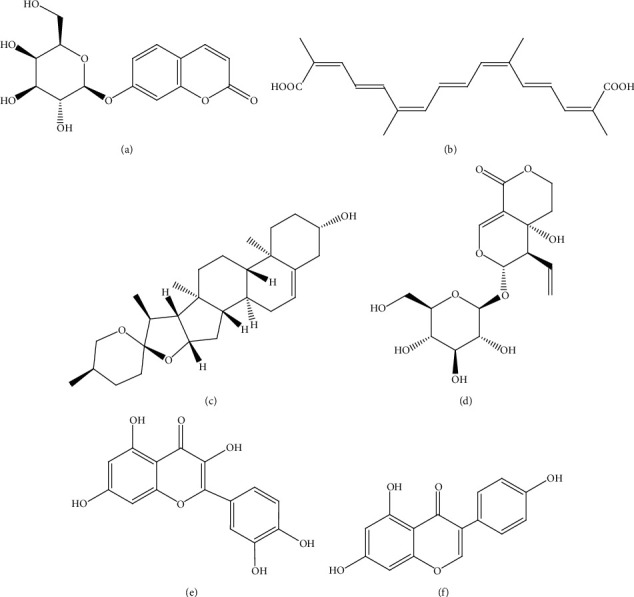
Structures of metabolites (a) umbelliferone beta-D-galactopyranoside (**18**), (b) saffron (**19**), (c) diosgenin (**20**), (d) swertiamarin (**21**), (e) quercetin (**22**), and (f) genistein (**23**) isolated from medicinal plants targeting *β-*cells.

**Figure 8:**
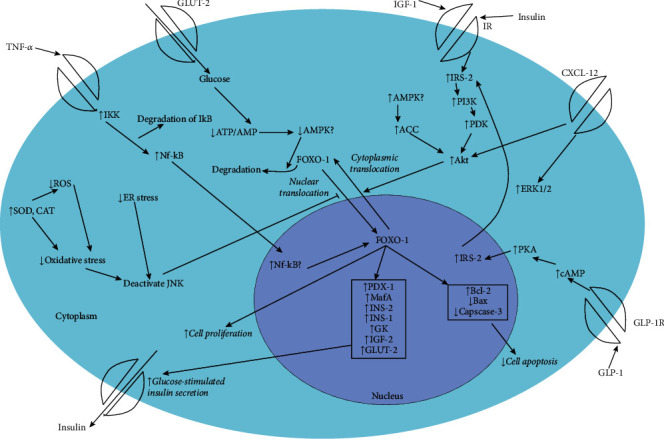
Summary of mechanisms by which natural products improve *β-*cell function and regeneration. TNF-*α*, tumor necrosis factor-*α*; NF-*κ*B, nuclear factor *κ*B; IkB, inhibitor of NF-*κ*B; IKK, I*κ*B kinase; ATP, adenosine triphosphate; AMP, adenosine monophosphate; AMPK, AMP-activated protein kinase; FOXO-1, forkhead box O1; GLUT-2, glucose transporter-2; IGF-1, insulin-like growth factor-1; IR, insulin receptor; IRS-2, insulin receptor substrate-2; PI3K, phosphoinositide 3-kinase; PDK, phosphoinositide-dependent protein kinase; Akt, protein kinase B; ACC, acetyl-CoA carboxylate; CXCL-12, C-X-C motif chemokine 12; ERK 1/2, Extracellular signal-regulated protein kinase 1/2; PKA, protein kinase A; cAMP, cyclic AMP; GLP-1R, Glucagon-like peptide-1 receptor; SOD, superoxide dismutase; CAT, catalase; ROS, reactive oxygen species; PDX-1, pancreatic duodenal homeobox-1; MafA, v-maf musculoaponeurotic fibrosarcoma oncogene family protein A; INS-1, insulin-1; INS-2, insulin-2; GK, glucokinase; IGF-2, insulin-like growth fator-2; Bcl-2, B-cell lymphoma-2; Bax, Bcl-2-associated X, caspase-3, cysteinyl aspartate specific proteinase-3.

**Figure 9:**
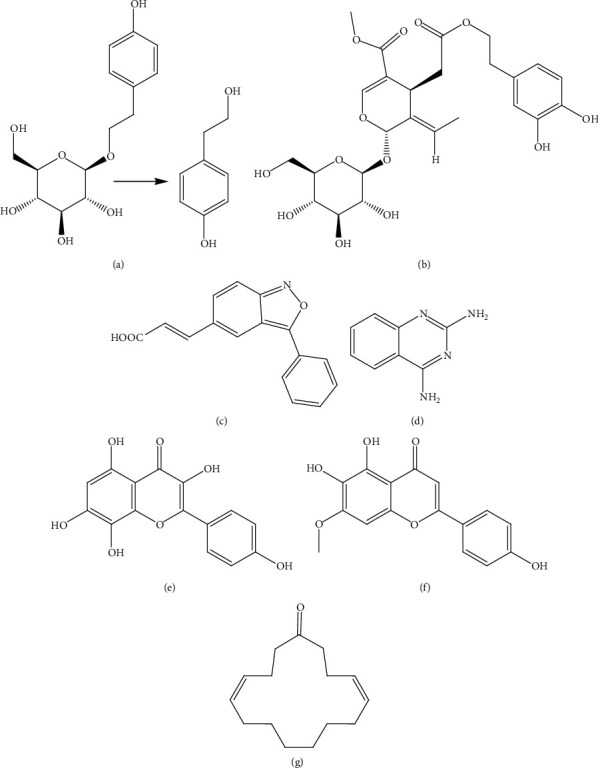
Compounds targeting *β-*cells as evidenced by SAR studies: (a) *p*-tyrosol (**31**), (b) oleuropein (**32**), (c) (*E*)-3-(3-phenylbenzo[c]isoxazol-5-yl) acrylic acid (**33**), (d) 2,4-diaminoquinazoline (**34**), (e) herbacetin (**35**), (f) sorbifolin (**36**), and (g) (4*Z*, 12*Z*)-cyclopentadeca-4,12-dienone (**37**).

**Figure 10:**
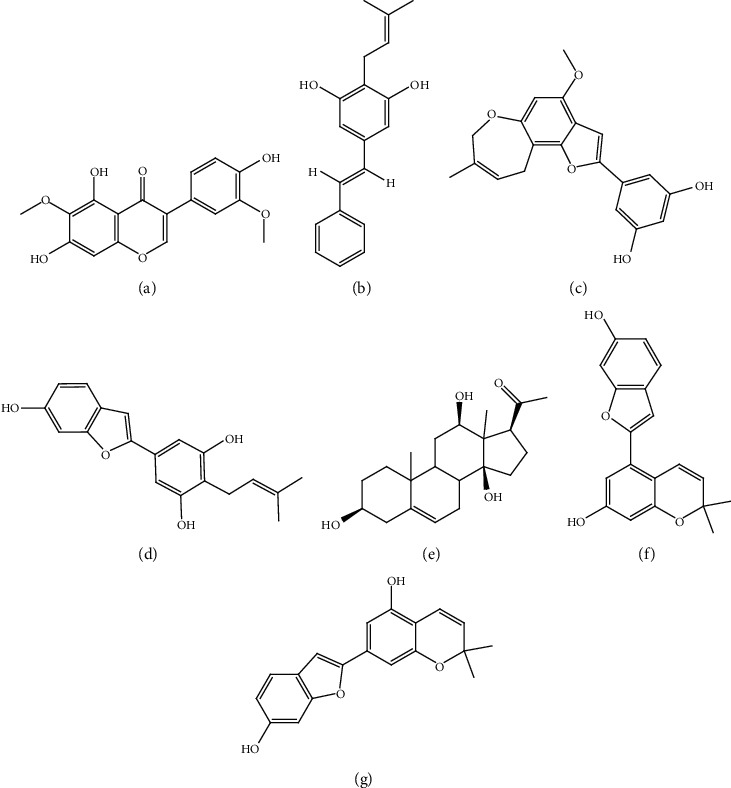
Structures of metabolites isolated from *Morus alba* Linne targeting *β-*cells as evident by SAR studies. (a) Iristectorigenin A (**38**), (b) 4-prenylresveratrol (**39**), (c) moracin H (**40**), (d) moracin C (**41**), (e) isoramanone (**42**), (f) moracin E (**43**), and (g) moracin D (**44**).

SAR studies reported on several other compounds are also noteworthy to identify pharmacophores targeting *β-*cells. A SAR study on oleuropein (**32**) (Figure 9) (a secoiridoid glycoside) isolated from leaves of *Olea europaea* (Family: Oleaceae) reported that 3-hydroxytyrosol moiety of oleuropein is the main entity responsible for amyloid inhibition. However, the entire structure scaffold of the molecule was found to be needed for its glucose-stimulated insulin secretion effect [150]. SAR study of a cinnamic acid derivative, (*E*)-3-(3-phenylbenzo[c]isoxazol-5-yl)acrylic acid (**33**) (Figure 9), with *β-*cell regeneration ability has shown the presence of both carboxylate group and the intact isoxazolyl ring required for its *β-*cell regeneration ability via cAMP/PKA/mTOR signaling pathway by inhibiting IKK and Ikb selectively in *β-*cells [151]. SAR optimization of a 2,4-diaminoquinazoline (**34**) (Figure 9) compound showed antiapoptotic action and increased insulin secretion which were mediated by regulating genes related to signaling pathways of caspase-3 and PDX1/MafA, respectively. These effects were optimum when 4-methoxy and benzylamine groups are present in the molecule [152]. Apart from the above, attempts have been made to identify potential *β-*cell targets of isolated compounds from medicinal plants through molecular docking studies. Data of molecular docking studies could be used in selecting potential antidiabetic agents for further *in vivo*/*in vitro* investigations. Herbacetin (**35**) (Figure 9) and sorbifolin (**36**) (Figure 9) isolated from *Ficus* species are capable of binding to amino acid residues Leu1002, Met1079, and Asp1150 of insulin receptor [153]. Glycogen synthase kinase-3 (GSK-3) is an important target in the regulation of *β-*cell mass and regeneration. Docking study on (4*Z*, 12*Z*)-cyclopentadeca-4,12-dienone (**37**) (Figure 9) isolated from *Grewia hirsuta* against target protein GSK-3 has shown that the oxygen atom of the isolated compound reacts with LYS-85 residue of GSK-3 and forms hydrogen bond through a strong hydrophobic interaction [154]. Quercetin (**22**) (Figure 6), iristectorigenin A (**38**), 4-prenylresveratrol (**39**), moracin H (**40**), moracin C (**41**), isoramanone (**42**), moracin E (**43**), and moracin D (**44**) (Figure 10) isolated from leaves of *Morus alba* Linne have shown good affinity to AKT-1 target protein [155].

Molecular docking and SAR studies conducted on compounds of different chemical categories are also reported, and these data could be useful in predicting activities of natural compounds with related structures. One such SAR study on benzamide derivatives reported that 3-(*N*-piperidinyl) methylbenzamide derivative protects *β-*cells against ER stress-induced dysfunction and death [156]. Virtual screening of thiadiazine compounds against dual-specificity tyrosine-phosphorylation-regulated kinase 1A (DYRK1A) enzyme has shown to be capable of inducing *β-*cell replication [157]. SAR optimization of harmine analogs revealed that derivatives of harmine with C-1 position substitutions act on DYRK1A promoting human *β*-cell proliferation at doses of 3–30 *μ*M [158].

## 5. Toxicity Concerns of Herbal Medicines Targeting *β-*Cells

Herbal medicines with a long history of use for DM are considered to be safe for therapeutic applications. Single herbal extracts and polyherbal mixtures as used by traditional medicine practitioners are generally considered to be time tested for safety and hence are allowed to be used directly in clinical applications [159]. However, fractionation or isolation of compounds from plant extracts may lead to toxicities [160]. The intention of coadministration of herbs or herbal compounds in traditional medicine practices is to have a synergistic therapeutic effect while ameliorating toxicities exerted by individual herbs. Scientific investigations in favor of combination therapy of different herbs to reduce toxicity exerted from individual herbs are also reported. Such attenuations in toxicities are usually caused by herb-herb interactions in polyherbal mixtures or mixtures of herbal compounds which leads to alterations of pharmacokinetics, i.e., absorption, distribution, metabolism, and elimination of constituents [161]. For example, Fructus Foeniculi (mature fruit of *Foeniculum vulgare* Mill.) and Fructus Meliae Toosendan (mature fruit of *Melia toosendan* Sieb. et Zucc) are two herbs used in combination in traditional Chinese medicine, and pharmacokinetic studies have revealed that Fructus Foeniculi attenuates the hepatotoxicity exerted by Fructus Meliae Toosendan via decreasing its absorption and increasing elimination thereby decreasing *in vivo* accumulation of the toxic constituent, toosendanin [162]. However, there is a possibility of occurring undesirable alterations of pharmacokinetics upon concomitant administration of different herbs especially in the case of introducing novel herbal mixtures. This could lead to unexpected toxicities by means such as accumulation of herbal constituents in the body or formation of toxic metabolites. For example, herbal compounds with cytochrome p450 inhibitory activity may diminish the metabolism of another coadministered herbal compound metabolizing via the same enzyme leading to toxicity from the latter [163]. For example, pyrrolizidine alkaloids are reported to exert hepatotoxicity and genotoxicity by metabolic activation via cytochrome p450 enzyme and modulate other hepatic metabolizing enzymes and hence could produce unexpected toxicities when coadministered with other herbal compounds with hepatic enzyme modulatory effect [164]. Most of the herbal medicines discussed in this review were tested using *in vitro* cellular models and rats for their toxicity and were proven to be safe. However, natural products usually have multiple bioactivities which may include unwanted/toxic effects when it comes to clinical applications. For example, asiatic acid found in this review also possesses anticancer activity where it inhibits antiapoptotic proteins causing cell death [165].

Extensive toxicity studies including *in vivo* acute and repeated dose toxicity, *in vitro* cytotoxicity, genotoxicity, carcinogenicity, teratogenicity, and drug interaction studies are required to ensure the safety of traditional herbal medicines [166]. Hence, in-depth long-term toxicity studies using *in vivo* models are warranted to ascertain the safety of herbal medicines as therapeutics. Apart from that, contamination of herbal medicines with heavy metals such as lead, arsenic, mercury, and cadmium which may lead to toxicity in consumers is a matter of concern [167]. Hence, standardization of herbal medicines using limited tests for these heavy metals is also needed.

## 6. Challenges and Future Perspectives

Herbal medicines due to their multitasking ability and low toxicity (at prescribed doses) compared to synthetic medicines could be useful in addressing therapeutic needs of multifactorial diseases such as DM that have not yet been met by synthetic medicines. There is a vast knowledge of herbal medicines with antidiabetic effects residing among different nations and ethnic groups. Retrieval of this information would pave the way to develop better therapeutic agents for DM particularly targeting the *β-*cell functions.

An increasing trend of investigating herbal medicines targeting pancreatic *β-*cells as a preventive/treatment strategy for DM is observed in recent years. However, long-term *in vivo/in vitro* investigations on determining biological effects and toxicities as well as elucidating molecular and cellular mechanisms are needed to ensure the safety and efficacy of these products.

The involvement of *β-*cells in disease pathogenesis and identification of molecular and cellular targets of *β-*cells for effective treatment of DM has also extensively been studied. Hence, elucidation of SAR of natural bioactive compounds targeting *β-*cells is important in identifying/developing lead compounds in the development of novel therapeutic options against DM.

The low aqueous solubility, poor absorption leading to low bioavailability, unpleasant taste, and lack of standardization are major limitations of herbal medicines. Encapsulation of herbal medicines into biocompatible nanomaterials is gaining attention to overcome these limitations. An attempt has been made to nanoencapsulated curcumin in polylactide-co-glycolide. Oral administration of 50 mg/kg nanocurcumin followed by intraperitoneal injection of STZ (60 mg/kg) showed *β-*cell preservation as evident by TUNEL and H&E staining of pancreatic tissue, an effect which was not observed in the plain curcumin-treated group [168]. Hence, nanoformulations could be useful in the targeted delivery of bioactive compounds to *β-*cells with improved solubility and increased bioavailability.

## 7. Conclusions

A number of single herbal extracts, polyherbal mixtures, and isolated compounds from plant extracts as well as combinations of natural bioactive compounds targeting pancreatic *β-*cells have been investigated as preventive/treatment strategies for DM in recent years. Many herbal products reported an improvement of *β-*cell function as observed through HOMA-*β*. Pancreatic *β-*cell regeneration is observed prominently in terms of increase in size and number of pancreatic islets, the number of insulin-secreting cells, and the number of proliferating *β-*cells in histopathological/immunohistochemical studies. Increasing *β-*cell mass via the expression of genes/proteins related to antiapoptotic actions and *β-*cell neogenesis/proliferation was identified as a mechanism of *β-*cell regeneration in some herbal medicines. Increasing glucose-stimulated insulin secretion via activating GLUT-2 receptors and/or increasing intracellular Ca^2+^ levels was also observed upon treatment with some herbal medicines. Most of the studies suggested that reduction of oxidative stress inflammation by natural products has protective effects on *β-*cells. Furthermore, the herbal medicines acted on various insulin signaling pathways by regulating the expression of different receptors and transcriptional factors. However, there are knowledge gaps and unexplored avenues on mechanisms of action of herbal medicines on pancreatic *β-*cells, their SAR, and toxicities which should be further investigated in order to develop effective therapies against the management of DM.

## Figures and Tables

**Figure 7:**
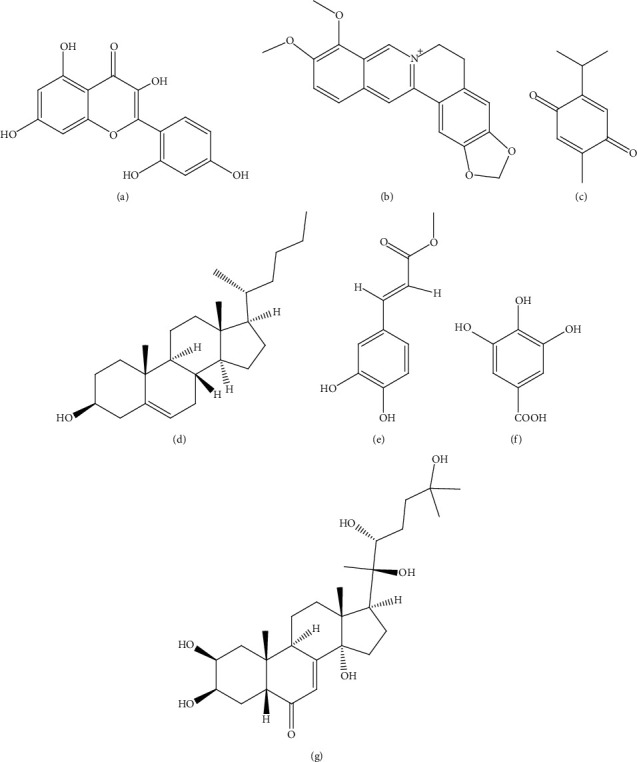
Structures of metabolites (a) morin (**24**), (b) berberine (**25**), (c) thymoquinone (**26**), (d) nymphayol (**27**), (e) methyl caffeate (**28**), (f) gallic acid (**29**), and (g) 20-hydroxyl ecdysone (**30**) isolated from medicinal plants targeting *β-*cells.

**Table 1:** Investigations on experimental drugs derived from plant sources for their ability to improve *β-*cell regeneration.

Experimental drug	Experimental system	Dose	*β-*Cell regeneration mechanism	Reference
Harmine	C57B6 mice with partial pancreatectomy and human islet transplantation	10 mg/kg	*β-*Cell proliferation	[14]
	Rat insulinoma (Ins1 823/13) and human hepatoma (HepG2) cell lines	1–15 *μ*M	*β-*Cell proliferation	

*γ*-Aminobutyric acid	Wild-type transgenic mice induced with streptozotocin (60 mg/kg)	250 *μ*g/kg	*β-*Cell regeneration via differentiation of *α*-cells to *β-*cells	[16]

Artemether	Mouse *β-*cell line (Min6) induced with doxycycline (1 *μ*g/mL) for overexpression of ARX transcription factor	10 *μ*M	ARX repression thereby increase *β-*cell turnover and conversion of *α*-cells to *β-*cells	[17]
	Zebrafish larvae induced with dimerizer AP20187 (2 *μ*M)	5 *μ*M	*β-*Cell proliferation and conversion of *α*-cells to *β-*cells	
	Sprague–Dawley rats induced with streptozotocin (60 mg/kg)	20–200 mg/kg	*β-*Cell proliferation and conversion of *α*-cells to *β-*cells	

**Table 2:** Brief summary of medicinal plant extracts targeting *β-*cells via undiscovered mechanisms.

Plant	Family	Part of the plant used	Type of extract	Secondary metabolite of interest	Experimental model	Effects on *β-*cells	Reference
*Abrus precatorius* L.	Fabaceae	Leaves	Ethanol	NM	Sprague–Dawley rats induced with NA (110 mg/kg, i.p.) and STZ (60 mg/kg, i.p.)	Recovery of damaged pancreatic *β-*cells increase in median cross-sectional area of pancreatic islets	[63]

*Aegle marmelos* (L.) Corr. Serr.	Rutaceae	Leaves	95% ethanol	NM	Albino rats induced with STZ (55 mg/kg, i.p.)	Protection of pancreatic *β-*cells from oxidative stress	[64]

*Aegle marmelos* (L.) Corr. Serr.	Rutaceae	Bark	Methanol	Aegelin (alkaloid) and lupeol (triterpenoid)	Wistar rats induced with STZ (60 mg/kg, i.p.)	Regeneration of *β-*cells Increase in insulin producing *β-*cells	[65]

*Aloe vera* (L.) Burm. f.	Asphodelaceae	Leaves	Aqueous	NM	Wistar rats induced with STZ (30 mg/kg, i.p.)	Restoration of pancreatic islet mass	[66]

*Althaea officinalis* L.	Malvaceae	Seeds	Methanol	NM	Wistar rats induced with STZ (90 mg/kg, i.p.)	Increase in islet number and diameter	[67]

*Antidesma bunius* L.	Phyllanthaceae	Leaves	Methanol	NM	Albino mice induced with alloxan (150 mg/kg, i.p.)	Improvement in pancreatic structure Regeneration of the *β-*cells	[68]

*Aronia melanocarpa* (Michx.) Elliott	Rosaceae	Fruits	Aqueous	NM	RINm5F insulinoma cells	Protection of pancreatic *β-*cell from oxidative damage	[69]

*Artocarpus altilis* (Parkinson) Fosberg	Moraceae	Leaves	Ethanol	NM	Albino rats induced with STZ (50 mg/kg, i.p.)	Increase in insulin expression in *β-*cells	[70]

*Aster spathulifolius* Maxim.	Asteraceae	Whole plant	Ethanol	3, 5−/4, 5-Dicaffeoylquinic acid and chlorogenic acid	C57BL/KsJ-db/db diabetic mice	Upregulation of insulin production by increasing pancreatic *β-*cell mass	[71]

*Atriplex polycarpa* (Torr.) S. Watson	Amaranthaceae	Stem bark	Aqueous	Alkaloids and phenolics (flavonoids)	ICR mice induced with alloxan (150 mg/kg, i.p.)	Regeneration of *β-*cells	[72]

*Azadirachta indica* A. Juss. *Bougainvillea spectabilis* Willd.	Meliaceae Nyctaginaceae	Leaves	Chloroform Aqueous, Methanol	NM	Swiss mice induced with STZ (120 mg/kg, i.p.)	Regeneration of *β-*cells	[73]

*Calotropis gigantean* (L.) W.T. Aiton	Apocynaceae	Flowers	Chloroform	NM	Wistar rats induced with STZ (40 mg/kg, i.p.)	Protection of *β-*cells from oxidative stress by decreasing pancreatic thiobarbituric acid-reactive substances (TBARS) levels increasing the SOD, CAT, and GSH levels	[74]
*Canscora decussata* (Roxb.) Schult.	Gentianaceae	Whole plant	Methanol	NM	Rabbits induced with alloxan (150 mg/kg, iv)	Regeneration of *β-*cells	[75]

*Carica papaya* L. *Pandanus amaryllifolius* Roxb.	Caricaceae Pandanaceae	Leaves	Ethanol	NM	Mice induced with STZ (60 mg/kg, i.p.)	Regeneration of *β-*cells	[76]

*Cassia occidentalis* Linn.	Fabaceae	Whole plant	Ethanol	NM	Wistar rats induced with alloxan (120 mg/kg, i.p.)	Regeneration of *β-*cells	[77]

*Chiliadenus iphionoides* (Boiss. and Blanche) Brullo	Asteraceae	Aerial parts	95% ethanol	NM	Pancreatic RIN-5F cells	Increase in insulin secretion	[78]

*Citrullus colocynthis* (L.) Schrad.	Cucurbitaceae	Seeds	Petroleum ether	NM	Wistar rats induced with alloxan (65 mg/kg, i.p.)	Partial preservation/restoration of pancreatic *β-*cell mass	[79]

*Clitoria ternatea* L.	Fabaceae	Aerial parts	Ethanol	Polyphenols	Wistar rats induced with alloxan (45 mg/kg, sc)	Regeneration of *β-*cells	[80]

*Crassocephalum crepidioides* (Benth.) S. Moore	Asteraceae	Aerial parts	80% methanol	NM	Wistar rats induced with alloxan (150 mg/kg, i.p.) INS-1 cells	Increase in the percentage of *β-*cells Protection of pancreatic *β-*cell from alloxan-induced apoptosis and from intracellular reactive oxygen species (ROS) accumulation	[81]

*Coccinia grandis* (L.) Voigt	Cucurbitaceae	Leaves	Aqueous	NM	Wistar rats induced with alloxan (150 mg/kg)	Regeneration of *β-*cells	[82]

*Costus igneus* N.E.Br.	Costaceae	Rhizome	Ethanol	Quercetin and kaempferol (flavonoids)	Albino rats induced with STZ (40 mg/kg, i.p.)	Regeneration of *β-*cells	[83]

*Curcuma longa* L.	Zingiberaceae	Roots	Hydroalcoholic extract	NM	RINm5F cell line induced with STZ (2 mM)	Inhibition of MDA release Inhibition of *β-*cell apoptosis	[84]

*Dacryodes edulis* (G. Don) H.J. Lam	Burseraceae	Fruit	Hexane	NM	Albino rats induced with alloxan (150 mg/kg, i.p.)	Restoration of the damaged pancreatic *β-*cell architecture	[85]

*Dacryodes edulis* (G. Don) H.J. Lam	Burseraceae	Leaves	Ethanol	Phenolics-gallic acid, vanillic acid, vanillin, and (−)-epicatechin	Albino rats induced with 10% fructose and STZ (40 mg/kg, i.p.)	Increase in HOMA-*β* Improvement in pancreatic morphology	[86]

*Eriobotrya japonica* (Thunb.) Lindl.	Rosaceae	Leaves	Aqueous	Cinchonain Ib	INS-1 cell	Increase in insulin secretion	[87]

*Eurycoma longifolia* Jack	Simaroubaceae	Root	NA	NM	db/db diabetic mice	Proliferation of *β-*cell and increase in *β-*cell number and PDX1 expression	[88]

*Ficus carica* L.	Moraceae	Leaves	Ethyl acetate	NM	Wistar rats induced with HFD and STZ (40 mg/kg, i.p.)	Protection of *β-*cells from oxidative stress Improvement of OGTT and ITT	[89]

*Gastrodia elata* Blume	Orchidaceae	Whole plant	Aqueous	NM	Sprague–Dawley rats induced by 90% pancreatectomy	Induction of hypothalamic insulin signaling Increase in mass of *β-*cells by potentiating proliferation and decreasing apoptosis	[90]

*Gossypium herbaceum* L.	Malvaceae	Seeds	Ethanol	NM	Rabbits induced with alloxan-induced (100 mg/kg, iv) diabetic rabbits	Protection of *β-*cells from oxidative stress	[91]

*Gmelina arborea* Roxb.	Verbenaceae	Stem bark	Aqueous	NM	Wistar rats induced with alloxan (150 mg/kg, i.p.) Wistar rats induced with STZ (65 mg/kg, i.p.)	Regeneration of *β-*cells Regeneration of *β-*cells	[82, 92]

*Gymnema montanum* (Roxb.) Hook.f. var. montanum	Apocynaceae	Leaves	Ethanol	NM	HIT-T15 *β-*cell line	Protection of pancreatic *β-*cells from alloxan-induced oxidative stress	[93]

*Gymnema sylvestre* (Retz.) Schult.	Apocynaceae	Leaf and callus	Methanol	Gymnemic acid	Wistar rats induced with alloxan (100 mg/kg, i.p.)	Regeneration of *β-*cells	[94]

*Hibiscus sabdariffa* L.	Malvaceae	Calyx	Methanol	NM	Wistar rats induced with STZ (80 mg/kg, i.p.)	Improvement of the volume of the pancreatic islets and the numerical density of *β-*cell (number of *β-*cells per unit area of islet)	[95]

*Hypoxis argentea* Harv. ex Baker	Hypoxidaceae	Corms	Aqueous	NM	INS-1 cells	A significant (*p* < 0.001) increase in total INS-1 cell numbers	[96]

*Ichnocarpus frutescens* (L.) W.T.Aiton	Apocynaceae	Leaves, stem, and flowers	Methanol	NM	Wistar rats induced with NA (230 mg/kg, i.p.) and STZ (65 mg/kg, i.p.)	Regeneration of *β-*cells	[97]

*Khaya senegalensis* (Desr.) A. Juss.	Meliaceae	Root	Butanol fraction of ethanol extract	NM	Sprague–Dawley rats induced with fructose (10%) and STZ (40 mg/kg, i.p.)	Improvement in of HOMA-*β*	[98]

*Laurus nobilis* L.	Lauraceae	Leaves	Ethanol	NM	Wistar rats induced with STZ (70 mg/kg, i.p.)	Regeneration of pancreatic islets	[99]

*Leea macrophylla* (Roxb.) ex Hornem	Vitaceae	Root	Ethanol	NM	Wistar rats induced with STZ (60 mg/kg, i.p.)	Reduction of oxidative stress Repair of *β-*cell damage	[100]

*Mangifera indica* L.	Anacardiaceae	Leaves	Alcohol	NM	Swiss albino mice induced with alloxan (150 mg/kg, i.p.)	Regeneration of *β-*cells	[101]

*Momordica charantia* L.	Cucurbitaceae	Fruit pulp	Ethanol	NM	Wistar rats induced with STZ (100 mg/kg, i.p.)	Improvement of HOMA-*β* Increase in islet size, total *β-*cell area and number of insulin-positive *β-*cells	[102]

*Momordica charantia* L.	Cucurbitaceae	Fruit	NA	NM	Wistar rats induced with HFD and STZ (40 mg/kg, i.p.)	Regeneration of *β-*cells	[103]

*Momordica charantia* L.	Cucurbitaceae	Fruit	Aqueous	NM	Albino rats induced with STZ (55 mg/kg, i.p.)	Regeneration of *β-*cells	[104]

*Moringa oleifera* Lam.	Moringaceae	Leaves	Aqueous	NM	Wistar rats induced with alloxan (120 mg/kg, i.p.)	Regeneration of damaged hepatocytes and pancreatic *β-*cells	[105]

*Nypa fruticans* Wurmb.	Arecaceae	Vinegar	Aqueous	NM	Sprague–Dawley rats induced with STZ (55 mg/kg, i.p.) RIN-5F cell culture	Increase in insulin production Stimulatory effect on insulin release at a basal glucose concentration (1.1 mM)	[106]

*Otostegia persica* Boiss	Lamiaceae	Aerial parts	Methanol	NM	C187 pancreatic *β-*cells	Increase in GSIS	[107]

*Parkia biglobosa* (Jacq.) G. Don	Fabaceae	Leaves	Butanol fraction	NM	Sprague–Dawley rats induced with STZ (40 mg/kg, i.p.)	Improvement of HOMA-*β*	[108]

*Phyllanthus emblica* L.	Phyllanthaceae	Fruits	Hydroalcoholic extract	NM	RINm5F cell line induced with STZ (2 mM)	Inhibition of MDA release Inhibition of *β-*cell apoptosis	[84]
*Prosopis cineraria* (L.) Druce	Fabaceae	Pods	Ethanol	NM	Albino rats induced with high sucrose diet and dexamethasone (1.5 mg/kg, i.p.)	Increase in HOMA-*β-*increase in pancreatic cell proliferation	[109]

*Pseuduvaria macrophylla* (Oliv.) Merr.	Annonaceae	Stem bark	Methanol and chloroform	Polyphenols	Sprague–Dawley rats induced with NA (210 mg/kg, i.p.) and STZ (55 mg/kg, i.p.)	Reduction of oxidative stress Downregulation of the levels of proinflammatory cytokines	[110]

*Spondias pinnata* (Linn. f.) Kurz	Anacardiaceae	Stem bark	Aqueous	NM	Wistar rats induced with alloxan (150 mg/kg)	Regeneration of *β-*cells	[82]

*Spondias pinnata* (Linn. f.) Kurz.	Anacardiaceae	Stem bark	Aqueous	NM	Wistar rats induced with STZ (65 mg/kg, i.p.)	Islet cell regeneration as noted by the increase in insulin-secreting *β-*cells and increase in islet profile diameter in the pancreas	[111]

*Syzygium densiflorum* Wall. ex Wight and Arn	Myrtaceae	Fruits	Methanol	NM	Wistar rats induced with NA (110 mg/kg, i.p.) and STZ (65 mg/kg, i.p.)	Regeneration of *β-*cells	[112]

*Swertia macrosperma* C.B.Clarke	Gentianaceae	Whole plant	90% ethanol	Polyphenols	Wistar rats induced with high-fat-high fructose diet and STZ (35 mg/kg, i.p.)	Protection of pancreatic *β-*cells from oxidative stress Stimulation of insulin secretion from the remaining pancreatic *β-*cells	[113]

*Tamarindus indica* L.	Fabaceae	Seed coat	95% ethanol	Polyphenols	Wistar rats induced with alloxan (120 mg/kg, i.p.)	Protection of pancreatic *β-*cells from oxidative stress	[114]

*Tamarix stricta* Boiss.	Tamaricaceae	Aerial parts	70% ethanol	NM	Pancreatic RIN-5F cells Albino BALB/c mice induced with HFD and STZ (40 mg/kg, i.p.)	Preservation of *β-*cells	[115]

*Teucrium polium* L.	Lamiaceae	Methanol	Methanol	Rutin and apigenin (flavonoids)	Isolated rat pancreatic islets	Increase in insulin release	[116]

*Tinospora cordifolia* (Thunb.) Miers	Menispermaceae	Stem	Hexane, ethyl acetate, and methanol	NM	Albino rats induced with STZ (55 mg/kg, i.p.)	Regeneration of *β-*cell	[117]

*Tinospora cord folia* (Thunb.) Miers	Menispermaceae	Stems	Hydroalcoholic extract	NM	RINm5F cell line induced with STZ (2 mM)	Inhibition of MDA release Inhibition of *β-*cell apoptosis	[84]

*Urena lobata* L.	Malvaceae	Leaves	Aqueous	NM	Sprague–Dawley rats induced with high fructose diet and STZ (25 mg/kg, i.p.)	Improvement in the structure and function of *β-*cells Prevention of degradation of GLP-1 by inhibition of DPP-4 activity	[118]

*Urtica dioica* L.	Urticaceae	Leaves	90% ethanol	NM	Wistar rats induced with STZ (50 mg/kg, i.p.) RIN-5F cells	Regeneration of *β-*cells and reduction of *β-*cell damage Increase in insulin secretion	[119]

*Vitex doniana* Sweet.	Verbenaceae	Leaves	Aqueous and ethanol	NM	Albino rats induced with STZ (60 mg/kg, i.p.)	Regeneration of *β-*cells along with repair of *β-*cells	[120]

*Vitellaria paradoxa* C.F. Gaertn.	Sapotaceae	Barks	Aqueous	NM	Wistar rats induced with HFD and STZ (35 mg/kg, i.p.)	Increase in the size and number of islets in the pancreas	[121]

*Zingiber officinale* Roscoe	Zingiberaceae	Rhizome	96% ethanol and supercritical CO_2_ extracts	NM	INS-1 cells	Modulation of insulin release by interacting with serotonin (5-HT) receptor channel system	[122]

DM, diabetes mellitus; STZ, streptozotocin; NA, nicotinamide; i.p., intraperitoneal; iv, intravenous; sc, subcutaneous; HFD, high-fat diet; homeostatic model assessment-*β-*cell function HOMA-*β*; malondialdehyde, MDA; superoxide dismutase, SOD; neurogenin 3, Ngn3; natural killer cell transcription factor-related, gene family 6, locus 1, Nkx6.1; TBARS, thiobarbituric acid-reactive substances; CAT, catalase; GSH, reduced glutathione; ROS, reactive oxygen species; PDX1, pancreatic duodenal homeobox-1; OGTT, oral glucose tolerance test; ITT, GSIS, glucose-stimulated insulin secretion; GLP-1, glucagon-like peptide-1; DPP-4, dipeptidyl peptidase inhibitor-4; 5-HT, serotonin; NA, not applicable; NM, not mentioned.

**Table 3:** Plant secondary metabolites targeting *β-*cells via undiscovered mechanisms.

Plant source	Family	Isolated compound/s	Experimental model	Mode of action	Reference
*Aegle marmelos* Correa.—stem bark	Rutaceae	Umbelliferone *β*-D-galactopyranoside (**18**) (Figure 6)	Wistar rats with DM induced by STZ (60 mg/kg, i.p.)	Improvement of plasma insulin level	[123]

*Centella asiatica* L.	Apiaceae	Asiatic acid (**16**) (Figure 5)	GK rats with T2DM	Reduction of islet fibrosis Reversal of overexpressed fibronectin; a key protein related to islet fibrosis	[124]

*Crocus sativus* L.—flower	Iridaceae	Saffron (**19**) (Figure 6)	RIN-5F cells	Stimulation of insulin release	[125]

*Dendrobium huoshanense* C.Z. Tang and S.J. Cheng	Celastraceae	Polysaccharide	C57BL/6 mice with DM induced by HFD followed by STZ (100 mg/kg, i.p.)	Increase in *β-*cell mass Improvement of HOMA-*β*	[126]

*Dioscorea* species	Dioscoreaceae	Diosgenin (**20**) (Figure 6) (a phytosteroid sapogenin)	Albino rats with DM induced by STZ (40 mg/kg, i.p.)	Regeneration of *β-*cells	[127]

*Enicostemma* species Multiple edible plants	Gentianaceae	Swertiamarin (**21**) (Figure 6) (secoiridoid glycoside) quercetin (**22**) (Figure 6) (flavanoid) in combination	Albino rats with DM induced by STZ (50 mg/kg, i.p.)	Regeneration of pancreatic islets	[128]

*Hyoscyamus albus* L.—seeds	Solanaceae	Calystegines (polyhydroxylated alkaloids and imino-sugars)	Albino rats with DM induced by STZ (130 mg/kg, i.p.)	Regeneration of *β-*cells	[129]

*Momordica charantia* L.	Cucurbitaceae	Genistein (**23**) (Figure 6)	Wistar rats with DM induced by HFD followed by STZ (40 mg/kg, i.p.)	Regeneration of *β-*cells	[103]

Multiple plants	NA	Morin (**24**) (Figure 7) (flavonoid)	Albino rats with DM induced by STZ (50 mg/kg, i.p.)	Preservation of the normal histological appearance of pancreatic islets Preservation of insulin-positive *β-*cells	[130]

Multiple plants	NA	Berberine (**25**) (Figure 7) (alkaloid)	Wistar rats with DM induced by STZ (35 mg/kg, i.p.)	Increase in insulin expression Regeneration of *β-*cells Decrease in MDA and increase in SOD	[131]

*Nigella sativa* L.	Ranunculaceae	Thymoquinone (**26**) (Figure 7)	Wistar rats with DM induced by STZ (45 mg/kg, i.p.)	Improvement of the morphology of the pancreas	[132]

*Nymphaea stellate* Willd.—chloroform extract of flower	Nymphaeaceae	Nymphayol (**27**) (Figure 7) (A sterol)	Wistar rats with DM induced by STZ (55 mg/kg, i.p.)	Increase in the number of *β-*cell mass Increase in islet-like cell clusters in the islets of Langerhans	[133]

*Protorhus longifolia* (Bernh.) Engl.	Anacardiaceae	Lanosteryl triterpene, methyl-3*β*-hydroxylanosta-9,24-dien-21-oate	Sprague–Dawley rats induced with DM by HFD followed by STZ (30 mg/kg, i.p.)	Reduction of oxidative stress and inflammation Improvement of pancreatic structure	[134]

*Rosa canina* L.—fruits	Rosaceae	Oligosaccharide	Wistar rats with DM induced by STZ (60 mg/kg, i.p.)	Improvement of the structure of pancreatic *β-*cells and tissues Increase expression of Ngn3, Nkx6.1, and insulin	[135]

*Solanum torvum* Sw.—fruit	Solanaceae	Methyl caffeate (**28**) (Figure 7)	Albino rats with DM induced by STZ (55 mg/kg, i.p.)	Regeneration of *β-*cells	[136]

Terminalia bellirica (Gaertn.) Roxb.—fruit rind	Combretaceae	Gallic acid (**29**) (Figure 7)	Albino rats with DM induced by STZ (50 mg/kg, i.p.)	Regeneration of *β-*cells	[137]

*Vitex negundo* (Linn.)	Verbenaceae	20-OH ecdysone (**33**) (Figure 7)	Albino rats with DM induced by STZ (45 mg/kg, i.p.)	Regeneration of pancreatic islets	[138]

DM, diabetes mellitus; STZ, streptozotocin; i.p., intraperitoneal; HFD, high-fat diet; homeostatic model assessment-*β-*cell function HOMA-*β*; malondialdehyde, MDA; superoxide dismutase, SOD; neurogenin 3, Ngn3; natural killer cell transcription factor‐related, gene family 6, locus 1, Nkx6.1; NA, not applicable.

## Data Availability

No data were used to support this study.
